# Screening of Hydroxyapatite Biomaterials for Alveolar Augmentation Using a Rat Calvaria Critical-Size Defect Model: Bone Formation/Maturation and Biomaterials Resolution

**DOI:** 10.3390/biom12111677

**Published:** 2022-11-12

**Authors:** Cristiano Susin, Jaebum Lee, Tiago Fiorini, Ki-Tae Koo, Peter Schüpbach, Amanda Finger Stadler, Ulf ME Wikesjö

**Affiliations:** 1 Laboratory for Applied Periodontal & Craniofacial Research (LAPCR), Division of Comprehensive Oral Health, Adams School of Dentistry, University of North Carolina at Chapel Hill, Chapel Hill, NC 27599, USA; 2 Section of Periodontology, School of Dentistry, Federal University of Rio Grande do Sul, Porto Alegre 90000-000, Brazil; 3 Department of Periodontology and Dental Research Institute, School of Dentistry, Seoul National University, Seoul 110-460, Korea; 4 Schüpbach Ltd., 8800 Thalwil, Switzerland

**Keywords:** biocompatible materials, bone, alveolar bone grafting

## Abstract

Background: Natural (bovine-/equine-/porcine-derived) or synthetic hydroxyapatite (HA) biomaterials appear to be the preferred technologies among clinicians for bone augmentation procedures in preparation for implant dentistry. The aim of this study was to screen candidate HA biomaterials intended for alveolar ridge augmentation relative to their potential to support local bone formation/maturation and to assess biomaterial resorption using a routine critical-size rat calvaria defect model. Methods: Eighty adult male Sprague Dawley outbred rats obtained from a approved-breeder, randomized into groups of ten, were used. The calvaria defects (ø8 mm) either received sham surgery (empty control), Bio-Oss (bovine HA/reference control), or candidate biomaterials including bovine HA (Cerabone, DirectOss, 403Z013), and bovine (403Z014) or synthetic HA/ß-TCP (Reprobone, Ceraball) constructs. An 8 wk healing interval was used to capture the biomaterials’ resolution. Results: All biomaterials displayed biocompatibility. Strict HA biomaterials showed limited, if any, signs of biodegradation/resorption, with the biomaterial area fraction ranging from 22% to 42%. Synthetic HA/ß-TCP constructs showed limited evidence of biodegradation/erosion (biomaterial area fraction ≈30%). Mean linear defect closure in the sham-surgery control approximated 40%. Mean linear defect closure for the Bio-Oss reference control approximated 18% compared with 15–35% for the candidate biomaterials without significant differences between the controls and candidate biomaterials. Conclusions: None of the candidate HA biomaterials supported local bone formation/maturation beyond the native regenerative potential of this rodent model, pointing to their limitations for regenerative procedures. Biocompatibility and biomaterial dimensional stability could suggest their potential utility as long-term defect fillers.

## 1. Introduction

Implant dentistry increasingly has become a favored approach to replace missing or severely compromised teeth. However, rehabilitation using a bone-anchored dental implant prosthesis may become a challenge in cases lacking in bone volume and geometry, thus demanding bone augmentation. Although autologous bone grafts represent a benchmark or gold standard for bone augmentation procedures, implant dentistry to a great extent relies on a perceived efficacy of surrogates including cadaver-sourced allogeneic or xenogeneic bone biomaterials, polymeric or ceramic synthetic biomaterials, or combinations thereof, also including devices for guided bone regeneration and, lately, biologic amplifiers [1,2,3,4]. 

Natural (bovine-/equine-/porcine-derived) or synthetic hydroxyapatite (HA) biomaterials or varietals combined with ß-tricalcium phosphate (ß-TCP) appear to be the preferred technologies among clinicians for bone augmentation procedures in preparation for implant dentistry [5,6,7]. The minimum quality for a successful technology used in support of alveolar ridge augmentation is biocompatibility, i.e., the biomaterial elicits none or only a minimal inflammatory reaction while filling void spaces in bone. Additional important qualities include osteoconduction, i.e., the biomaterial/technology enhances the local osteogenic response, passively serving as a scaffold for enhanced bone formation, and thirdly osteoinduction, i.e., the biomaterial/technology induces de novo bone formation, this usually requiring a biologic amplifier [3,4,8]. Resolution and biodegradation/bioresorption of implanted biomaterials appear to be equally important qualities. Biodegradation/bioresorption per se may elicit inflammatory reactions, in turn compromising local bone formation [9,10]. Slowly or non-resorbing biomaterials may in fact obstruct rather than enhance local bone formation [11,12,13]. Slowly or non-resorbing biomaterials may impede the mechanical properties of bone including dental implant osseointegration [14,15]. Furthermore, slowly or non-resorbing biomaterials may become a nidus for infectious processes when exposed in surgical sites as well as processes associated with peri-implantitis. The objective of this study was to screen candidate HA biomaterials intended for alveolar ridge augmentation relative to their potential to support local bone formation/maturation and to assess biomaterial resolution, using one of the routine critical-size rat calvaria defect models. All biomaterials evaluated displayed biocompatibility. 

## 2. Materials and Methods

Male Sprague Dawley outbred rats, age 11–12 weeks, weight 325–375 g, obtained from an approved USDA licensed vendor, were used. The animals were maintained in an Association for Assessment and Accreditation of Laboratory Animal Care International-accredited facility. Housing, husbandry, and experimental manipulations were performed in accordance with the National Research Council’s Guide for the Care and Use of Laboratory Animals following a protocol approved for this study by the Institutional Animal Care and Use Committee, Augusta University. 

The animals were acclimatized for at least 7 days. They were single housed in plastic cages labeled with cage cards and fitted with ear tags for identification. The cages were housed in purpose-designed rooms air-conditioned with 10–15 air changes/h, and temperature (18–22 °C) and relative humidity (30–70%) monitored daily. A 12/12 h light/dark cycle was used. The animals had ad libitum access to water and a standard laboratory diet. This report was prepared following the ARRIVE guidelines for reporting animal research [16].

### 2.1. Agents and Biomaterials

This study compared six candidate biomaterials intended for alveolar ridge augmentation with an established reference (Bio-Oss^®^, Geistlich, Wolhusen, Switzerland) and sham-surgery (no biomaterial used) for their potential to enhance local bone formation/maturation and to assess biomaterial resolution using an 8 weeks healing interval (Table 1).

### 2.2. Study Design

Eighty animals randomized into groups of ten either received one of the candidate biomaterials, the sham-surgery empty control, or the Bio-Oss reference control. A study coordinator assigned biomaterials/controls to 5 animals at a time and repeated the assignment to another 5 animals the following day. The surgeons were masked to this specific protocol. Based on experience from previous studies, [17,18,19,20], it was estimated that a sample size of 10 animals/group was necessary to achieve 80% power to detect a mean difference of 30% in defect closure between experimental groups (defect closure: 30 ± 15% vs. 60 ± 30%). A two-sided two-sample t-test with significance level of 5% was used for the sample-size calculation.

### 2.3. Surgical Procedures

The animals were pre-medicated using buprenorphine (0.01–0.05 mg/kg SC) and meloxicam (2 mg/kg SC). Anesthesia was induced using ketamine hydrochloride (65 mg/kg IP) and isoflurane (5%) in an induction chamber. After induction, the skull of the animal was shaved and disinfected using a 2% chlorhexidine solution. Animals were then entered into the surgical theater, stabilized using a stereotaxic device (Stoelting Company, Wood Dale, IL, USA), fitted with an anesthesia nose cone, and draped. Isoflurane (1.0–3.0%/O2) was administered to maintain a surgical plane of anesthesia. 

Experienced surgeons (CS, JL, KTK, TF) performed all surgeries using a routine critical-size rat calvaria defect model [17,18,19,20]. Using aseptic routines, a 3 cm midline incision was made through the skin along the sagittal suture of the skull. Soft tissues and periostea were elevated and reflected. Under saline irrigation, a critical-size, ø8-mm, through–through, osteotomy defect centered over the sagittal suture between lambda and bregma was created using an ø8 mm diamond-coated trephine bur (Continental Diamond Tool Corporation, New Haven, IN, USA). Care was exercised to leave the dura intact. The defects were filled (approx. 75 µL) with designated candidate biomaterial, the Bio-Oss reference control or received sham-surgery (no biomaterial used). Particulate biomaterials were hydrated in sterile saline prior to implantation. A template was used to standardize the volume of all grafting materials (O7 × 1 mm). Then, a sterile precut 10 × 12 mm titanium mesh (Jeil Medical, Seoul, Korea) was placed to protect the defect site from compression. Titanium meshes were molded to fit over the calvaria defect. Finally, the full-thickness flaps were adapted and closed for primary intention healing using surgical staples (Disposable Skin Staples, TFX Medicad, London, UK) ensuring everted wound margins.

### 2.4. Post-Surgery Procedures

The animals received yohimbine hydrochloride (1.5 mg/kg SC) to reverse anesthesia, were placed in cages, warmed on a heating pad, and observed for distress until they were able to move about. Meloxicam (2 mg/kg, SC, q24 h, up to 3 days) was administered for pain control. Animals still exhibiting signs of pain received additional dose(s) determined by an attending veterinarian. Surgical staples were removed under anesthesia (isoflurane (5%) using an induction chamber) at 7–10 days. The animals were euthanized at 8 weeks post-surgery using a pentobarbital sodium/phenytoin sodium anesthetic overdose (Euthasol Solution, Virbac, Fort Worth, TX, USA) followed by bilateral thoracotomy as a secondary method. Calvaria biopsies were harvested carefully removing overlying soft tissues, and then immersed in a 10% buffered formalin solution.

### 2.5. Histotechnical Preparation

The calvaria biopsies were prepared for light microscopy using the “sawing and grinding” technique [21]. Briefly, specimens were dehydrated in a graded ethanol series and infiltrated with methyl methacrylate resin (Technovit 7200 VLC, Heraeus Kulzer, Hanau, Germany). Infiltrated specimens were polymerized using light polymerization (Exakt Apparatebau, Norderstedt, Germany). Polymerized blocks were sectioned using a bandsaw equipped with a diamond-coated band (Exakt Apparatebau, Norderstedt, Germany). The sections were ground to 30–50 µm thickness using a micro-grinding unit (Exakt Apparatebau, Norderstedt, Germany), stepwise polished using diamond pastes (Struers, Ballerup, Denmark), and stained using Sanderson’s RBS stain and counter-stained with acid fuchsin (Dorn & Hart Microedge, Villa Park, IL, USA). The most central section from each defect site (determined by the size of the defect) was used for the histopathologic and histometric analysis.

### 2.6. Histopathologic and Histometric Analysis

One experienced examiner (UW) conducted the histopathologic evaluation including observations of woven/lamellar bone formation, cortex formation, and evidence of biodegradation (erosion, fragmentation) or bioresorption (presence of osteoclast-like cells/scalloped margins) of any implanted biomaterial, fibrovascular red or fatty marrow, and eventual biomaterial displacement using polarized and incandescent light microscopy (EX 43, Olympus America, Melville, NY, USA). Two experienced examiners (CS, AFS) identified the landmarks and extent of bone formation using polarized and incandescent light microscopy (BX 51, Olympus America, Melville, NY, USA). One calibrated examiner (AFS) performed the histometric analysis using incandescent and polarized light microscopy (BX 51, Olympus America, Melville, NY, USA), a microscope digital camera system (DP73, Olympus America, Melville, NY, USA) and a PC-based image analysis software (cellSens^®^ Dimension 1.11 Digital Imaging Software, Olympus America, Melville, NY, USA). The following parameters were recorded for each defect:

- Defect width: distance between the defect margins;
- Defect closure: fraction (%) of accumulated length of new bone formation between the defect margins;
- Defect area: area of regeneration including newly formed bone, residual biomaterial and other tissue limited by the defect margins, and the titanium mesh;
- Defect fill: total area of newly formed bone between the defect margins;
- Bone area fraction: fraction (%) of newly formed bone within the defect area;
- Residual biomaterial: total area of residual biomaterial between the defect margins;
- Biomaterial area fraction: fraction (%) of residual biomaterial within the defect area.

The examiners did not have access to group coding during the histological analysis; however, masking was not effective for all groups due to some biomaterials featuring unique characteristics. Calibration was performed by measuring 10 samples twice 10 days apart.

### 2.7. Statistical Analysis

Statistical analysis was performed using statistical software (Stata 13.1 for Mac, Stata Corporation, College Station, TX, USA). Measures of central tendency (means and medians) and variability (standard deviations, 95% confidence intervals, percentiles, and range) were calculated and presented. The primary outcome of the study was linear defect closure, and the secondary outcomes bone area fraction and biomaterial area fraction. A one-way analysis of variance followed by pairwise comparisons using the Bonferroni correction was used to estimate *p*-values. Significance level was set at 5%. Examiner reliability for the histometric evaluation was assessed using the intraclass correlation coefficient (ICC). This coefficient ranges between 0 and 1 and values close to 1 mean high reliability. The ICC was >0.95 for all parameters measured.

## 3. Results

### 3.1. Clinical Observations

Surgeries followed established routines. Healing events were generally unremarkable. Three animals succumbed in the immediate post-surgery sequel, eight animals showed slow recovery and were euthanized on veterinary indications (these animals were replaced). The defect sites in three additional animals became exposed, however, without need for veterinary intervention (these defect sites were excluded from the histological analysis).

### 3.2. Histopathologic Observations

**Figure 1:**
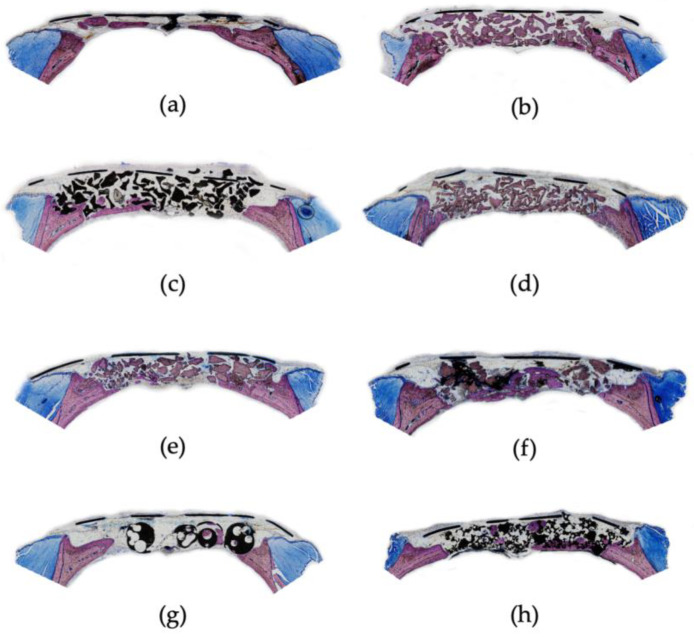
Photomicrographs of bone formation at defect sites receiving control (sham-surgery/Bio-Oss) and candidate biomaterials following an 8 wk healing interval: (**a**) sham-surgery, (**b**) *Bio-Oss,* (**c**) *Cerabone*, (**d**) *DirectOss* (**e**) *403Z013*, (**f**) *403Z014*, (**g**) *Ceraball*, and (**h**) *Reprobone.* Original magnification 4×.

The 8 weeks specimens showed limited lamellar bone formation variably extending from the defect margins for all candidate and control variations (Figure 1). Hematomas were observed in most groups. Seroma formation was not observed. The biocompatible particulate bovine-sourced HA candidate and control biomaterials resided in fibrovascular tissue without evidence of inflammatory reactions, or biodegradation/bioresorption (osteoclast-like cells, scalloped borders, and fragmentation). In contrast, the biocompatible synthetic HA/ß-TCP composites showed fragmentation/erosion suggestive of biodegradation of at least the ß-TCP component. Red or fatty marrow was not observed.

#### 3.2.1. Sham-Surgery

Defect sites receiving sham-surgery showed variable lamellar bone formation egressing from the calvaria periosteum at the defect margins (Figure 1a). The rest of the defects comprised fibrovascular tissue.

#### 3.2.2. Bio-Oss

Defect sites randomized to receive Bio-Oss (Geistlich, Wolhusen, Switzerland), the bovine bone-sourced particulate HA reference control, showed lamellar bone formation limited to the defect margins occasionally engaging adjoining biomaterial particles (Figure 1b). The biocompatible Bio-Oss HA particles resided in fibrovascular tissue without evidence of inflammatory reactions or biodegradation/bioresorption.

#### 3.2.3. Cerabone

Defect sites receiving Cerabone (Botiss Biomaterials, Zossen, Germany), a bovine bone-sourced particulate HA, showed noteworthy lamellar bone formation including cortex formation in three sites, whereas the remainder of the defect sites showed limited bone formation (Figure 1c). The biomaterial generally remained unaltered (geometry/presence); however, apparent fragmentation may suggest evidence for biodegradation/erosion in seemingly absence of osteoclast-like or inflammatory cells.

#### 3.2.4. DirectOss

DirectOss (Implant Direct, Thousand Oaks, CA, USA), a bovine bone-sourced particulate HA biomaterial, was associated with occasional and limited bone formation (Figure 1d). The biomaterial remained intact sequestered in fibrovascular tissue without evidence of inflammatory reactions or biodegradation/bioresorption.

#### 3.2.5. 403Z013

Defect sites implanted with 03Z013 (Certech, Seneffe, Belgium), a particulate bovine bone-derived HA biomaterial, showed lamellar bone formation circumscribing the particulate HA biomaterial suggestive of osteoconduction (three sites), and one site showing cortex formation in part of the defect (Figure 1d). The HA biomaterial remained largely intact dispersed in fibrovascular tissue without overt evidence of biodegradation/bioresorption.

#### 3.2.6. 403Z014

Defect sites receiving 3Z014 (Certech, Seneffe, Belgium), a particulate bovine bone-derived HA ß-tricalcium phosphate (ß-TCP)-coated biomaterial, showed lamellar bone formation including cortex formation encompassing up to 2/3 of the defect width (three sites) (Figure 1e). The 3Z014 HA biomaterial, considerably variable in particle size, remained largely intact immersed in fibrovascular tissue without overt evidence of biodegradation/bioresorption.

#### 3.2.7. Ceraball

Ceraball (Medartis, München, Germany), an HA (50%)/ß-TCP (50%) micro ball construct, was associated with limited variable bone formation, the micro ball structures immersed in fibrovascular tissue (Figure 1g). Lamellar bone formation approached the biomaterial as well as appeared sequestered within the micro ball structures. The HA/ß-TCP micro ball construct showed advanced biodegradation/erosion of presumably the ß-TCP component without substantial evidence of associated inflammatory reactions, whereas the overall geometry of the micro ball structures remained intact without significant signs of biodegradation.

#### 3.2.8. Reprobone

Defect sites receiving Reprobone (Ceramisys, Sheffield, UK), a synthetic particulate HA (60%)/ß-TCP (40%) biomaterial, displayed lamellar bone/cortex formation emerging from the defect margins engaging larger parts of the biomaterial (three sites) as well as solitary islands of bone dispersed amongst the overall intact (geometry/presence) biomaterial suggestive of osteoconduction (Figure 1f). Scant fragmentation suggests that the biomaterial at least in part is eroding and that there is an absence of a significant cellular/inflammatory response.

### 3.3. Histometric Analysis

Table 2 summarizes the results for the linear defect closure recordings. No statistically significant differences were observed among experimental groups, with sham-surgery displaying the highest (39.8%) and DirectOss the lowest (14.5%) mean linear defect closure. Data variability was moderate as appreciated by the SD and percentiles, and high and low values were not uncommon. 

Table 3 shows bone area fraction results according to experimental group. The sham-surgery group showed significantly greater bone area fraction than all other groups (17.1%, *p* < 0.001). Limited bone formation was observed for all other groups; no significant differences among experimental groups and the Bio-Oss reference control were observed, with the mean bone area fraction ranging from 3.2% (DirectOss) to 7.2% (Reprobone). 

Table 4 presents the residual biomaterial area fraction according to experimental group. Cerabone (42.3%) showed a significantly greater percentage of residual biomaterial than all but one group—Reprobone (*p* < 0.01). Intermediately, Reprobone (34.3%), Ceraball (28.7%), and DirectOss (25.9%) exhibited a mean biomaterial area fraction ranging from 25% to 35%. The lowest biomaterial area fraction was observed for 403Z013 (21.8%), Bio-Oss (21.3%), and 403Z014 (17.7%). 

## 4. Discussion

The objective of the present study was to screen candidate HA biomaterials considered for alveolar ridge augmentation relative to their potential to support local bone formation/maturation and to assess biomaterial resolution using a routine critical-size rat calvaria defect model and an 8 weeks healing interval. Six particulate HA species—bovine HA (Cerabone, DirectOss, 403Z013) and bovine (403Z014) or synthetic HA/ß-TCP (Reprobone, Ceraball) constructs—were benchmarked to sham surgery and a market-leader bovine HA reference control (Bio-Oss). None of the candidate biomaterials supported bone formation beyond the innate regenerative potential of this model. 

The present study used one of our laboratory routine critical-size rat calvaria defect models and an 8 weeks healing interval [17,18,19,20]. The rat calvaria defect model is widely accepted for screening osteogenic, osteoconductive and osteoinductive technologies including particulate HA species such as herein, other osteoconductive conduits and biologic amplifiers, successful technologies qualifying for pivotal evaluation in large-animal-discriminating inlay/onlay intraoral models, and, ultimately, clinical settings [11,22,23,24,25,26]. Means to evaluate treatment effects in the rat calvaria defect model include radiography, microcomputed tomography, biomechanical testing, and histology [25,26]; the present study focused on histology. An 8 weeks healing interval is designated to capture bone formation/maturation and biomaterial resolution (biodegradation/bioresorption) and side-effects, while shorter intervals (2 and 4 weeks) provide insights on bone formation [20]. 

Mean linear defect closure in the sham-surgery control approximated 40% in the present study consistent with previous studies, with mean linear defect closure in the sham-surgery control ranging from 32% to 58% following an 8 weeks healing interval [17,18]. Mean linear defect closure for the Bio-Oss reference control approximated 18% compared with 15–35% for the candidate biomaterials without significant differences between control and candidate biomaterials. Bone area fraction was significantly greater (17%) for the sham-surgery control than for any of the candidate biomaterials or the reference control, with the bone area fraction ranging from 3.2% to 7.2%. In other words, although biocompatible, none of the candidate biomaterials nor the reference control enhanced local bone formation. This observation is consistent with that by others, showing slowly or non-resorbing bovine-sourced or synthetic HA species frustrating bone formation in extraction sockets in dogs [13], in rat calvaria defects as in the present study [11], when used with bone morphogenetic protein onlays for ridge augmentation in dogs [27], and as bone morphogenetic protein/HA inlays for sinus augmentation [28], or HA/collagen constructs for ridge preservation in humans [12]. Collectively, these studies point to a general trend also discerned in the present screening of candidate/novel HA biomaterials for alveolar ridge augmentation; that is, HA varietals per se, whether cadaver-sourced bone derivatives or of synthetic manufacture, do not enhance local bone formation. 

Uneventful biodegradation/bioresorption are important qualities of bone biomaterials whether considered a stand-alone technology or married to biologic amplifiers [29,30]. The biocompatible bovine-sourced HA reference or candidate biomaterials in the present study mostly resided in fibrovascular tissue without evidence of inflammatory reactions or associated biodegradation/bioresorption, while the synthetic HA/ß-TCP constructs showed fragmentation/erosion suggestive of biodegradation of presumably at least the ß-TCP component. These observations are consistent with that by others using rat calvaria defects or canine models for alveolar ridge preservation/augmentation in the presence/absence of biologic amplifiers reporting limited biodegradation/bioresorption of implanted HA biomaterials, in contrast to a gradual fragmentation/erosion/resorption for ß-TCP varietals [11,13,27,31]. In perspective, Hong et al. reported 5–6% extraction socket bone fill at 2 weeks for an HA and a ß-TCP biomaterial [13]. Fast forward, bone fill at 8 weeks amounted to 32% and 49% for the HA and ß-TCP biomaterials, respectively, with the HA biomaterial comprising approximately 50% of the socket area at 2 and at 8 weeks compared with 38% and 10%, respectively, for the gradually eroding/resorbing ß-TCP biomaterial. Altogether, these histologic studies document a biological reality that HA species offer limited, if any, bioconversion, which may have implications for the biomechanical properties of sites augmented for implant dentistry as well as render them vulnerable to infectious processes. 

## 5. Conclusions

In summary, none of the candidate HA biomaterials supported local bone formation/maturation beyond the native regenerative potential of this rodent model pointing to their limitations for regenerative procedures. Biocompatibility and biomaterial dimensional stability could suggest their potential utility as long-term defect fillers.

## Figures and Tables

**Table 1:** Controls and candidate biomaterials.

	Composition	Particle Size (mm)	Source
Sham-surgery	-	-	-
Bio-Oss	bovine HA	0.25–1.00	Geistlich, Wolhusen, Switzerland
Cerabone	bovine HA	0.50–1.00	Botiss Biomaterials, Zossen, Germany
DirectOss	bovine HA	0.25–1.00	Implant Direct, Thousand Oaks, CA, USA
403Z013	bovine HA	0.10–2.00	Certech, Seneffe, Belgium
403Z014	bovine HA with ß-TCP coating	0.10–2.00	Certech, Seneffe, Belgium
Ceraball	synthetic HA(50%)/ß-TCP(50%)	1.50	Medartis, München, Germany
Reprobone	synthetic HA(60%)/ß-TCP(40%)	0.50–1.00	Ceramisys, Sheffield, UK

**Table 2:** Linear defect closure (%).

Group		Mean		Percentiles			Range	
	N	Mean *	SD	Median	25	75	Min	Max
Sham-surgery	9	39.8A	28.2	33.8	18.3	57.7	3.2	81.1
Bio-Oss	10	17.7A	10.3	13.0	9.6	25.1	4.6	37.3
Cerabone	10	25.7A	22.2	22.4	7.1	32.6	4.4	72.7
DirectOss	10	14.5A	11.0	13.9	5.6	22.8	0.0	33.3
403Z013	8	21.6A	21.6	10.1	7.5	39.4	0.0	59.3
403Z014	8	30.4A	25.7	26.8	7.2	50.1	2.1	73.0
Ceraball	10	34.4A	24.1	34.5	14.7	50.1	7.1	82.1
Reprobone	10	34.6A	21.5	34.6	14.5	56.4	6.1	61.1

* Means followed by the same capital letters are not significantly different (*p >* 0.05).

**Table 3:** Bone area fraction (%).

Group		Mean		Percentiles			Range	
	N	Mean *	SD	Median	25	75	Min	Max
Sham-surgery	9	17.1A	10.4	17.5	12.4	22.9	0.4	31.6
Bio-Oss	10	3.8B	2.1	3.7	2.0	5.4	0.4	7.4
Cerabone	10	5.3B	7.2	3.4	1.9	4.0	0.8	25.2
DirectOss	10	3.2B	2.0	3.3	1.7	5.1	0.0	5.6
403Z013	8	4.0B	4.3	1.8	1.6	6.8	0.0	11.6
403Z014	8	6.3B	5.2	6.6	1.6	9.3	0.3	15.4
Ceraball	10	6.6B	5.7	5.4	2.2	9.3	0.9	19.9
Reprobone	10	7.2B	6.3	6.3	2.1	10.2	0.6	21.2

* Means followed by the same capital letters are not significantly different (*p >* 0.05).

**Table 4:** Biomaterial area fraction (%).

Group		Mean		Percentiles			Range	
	N	Mean *	SD	Median	25	75	Min	Max
Sham-surgery	9	0.0A	0	0	0	0	0	0
Bio-Oss	10	21.3BC	10.4	22.1	9.7	27.9	8.5	38.5
Cerabone	10	42.3E	12.7	42.7	32.1	52.5	18.8	58.7
DirectOss	10	25.9BC	5.3	27.1	21.5	29.2	18.1	34.2
403Z013	8	21.8BC	8.7	20.2	15.1	31.2	10.2	31.7
403Z014	8	17.7B	6.9	19.4	13.9	21.3	5.0	27.8
Ceraball	10	28.7CD	10.5	27.3	22.6	35.8	11.9	48.6
Reprobone	10	34.3DE	11.1	33.6	26.7	39.6	14.1	56.0

* Means followed by the same capital letters are not significantly different (*p* > 0.05); experimental groups were ranked according to their mean to facilitate group comparisons.

## Data Availability

Data may be available by request to the corresponding author.
